# Newcastle Disease in Backyard Poultry Rearing in the Northeastern States of India: Challenges and Control Strategies

**DOI:** 10.3389/fvets.2022.799813

**Published:** 2022-04-07

**Authors:** Kekunguu Puro, Arnab Sen

**Affiliations:** ICAR Research Complex for NEH Region, Umiam, India

**Keywords:** Newcastle disease, backyard poultry, Northeast India, vaccination, endemic, NDV

Newcastle disease (ND) or Ranikhet disease is an avian viral disease, highly contagious in nature affecting many domestic avian species and wild birds. The disease is caused by infections with virulent *avian avulavirus 1*, commonly known as Newcastle disease virus (NDV) and designated as avian paramyxovirus-1 (APMV-1). It causes high morbidity and mortality in naïve, poorly vaccinated (1) or non-vaccinated birds, while the production performance in vaccinated birds is affected too when infected with virulent strains.

## Virus and Host Range

The different parts of the world reported different genotypes of APMV-1 in different avian species. Though all NDVs are the members of AMPV-1. NDV has been classified into class I and class II viruses based on their genetic characteristics. The class I viruses have been mostly isolated from wild birds and are generally of low virulence and rarely found in poultry species. There is a wider genetic variability reported within class II viruses, and currently, there are 18 class II genotypes based on the whole genome or the variability of protein F (1, 2). An updated NDV classification and nomenclature system incorporate phylogenetic topology, genetic distances, branch support, and epidemiological independence (3).

### Sequence of the F0 Cleavage Site of ND Virus and Its Role in Virulence

The virulence of NDV isolates is primarily determined by the sequence at the F cleavage site from positions 112–116 (4). The difference in the cleavage site of both the vaccine strains used in this study is indicative of the difference in the virulence attribute of these strains, namely, lentogenic for strain F and mesogenic for strain R2B in corroboration with the pathogenicity test data (Table 1). During virus replication, ND virus particles are produced with inactive, precursor F glycoproteins, termed F0. For the virus particles to be infectious, the F0 must be cleaved into two portions: the F1 and F2 polypeptides. The cleavability of the F0 glycoprotein is directly related to the virulence of viruses *in vivo* (5). It has been postulated that the F0 glycoproteins of virulent ND viruses can be cleaved by proteases found in many tissues and organs. Infection with these viruses results in the spread of the virus throughout the chicken or embryo, which damage many tissues and organs. In contrast, ND viruses of low virulence are sensitive to trypsin-like proteases only, which restricts infection to only certain cell types in the chicken or embryo. The molecular studies of the particular site on the F0 glycoprotein that undergoes cleavage have shown that a major influence on the pathogenicity of ND viruses is the amino acid sequence around this site. Thus, most virulent ND viruses have the sequence:

**Table 1:** Pathotypes of Newcastle disease virus [Young et al. (7)].

**Pathotype**	**Description of disease**	**Clinical signs and post mortem lesions**
Viscerotropic velogenic	Acute lethal infection in chickens of all ages	Hemorrhagic lesions in the gastrointestinal tract
Neurotropic velogenic	Acute infection in chickens of all a ges; high mortality	Respiratory and nervous signs
Mesogenic	Less pathogenic with low mortality, usually in young chickens	Respiratory and nervous signs
Lentogenic	Mild, inapparent infection; deaths confined to young chickens	Respiratory signs
Asymptomatic enteric (avirulent)	Avirulent infection; no mortality	No signs or lesions

112R or K-R-Q-K or R-R^*^ F117.

Whereas the sequence of thermostable NDV strain I-2 is:

112R-K-Q-G-R^*^ L117.

The sequence for strain I-2 is unique among the lentogenic or avirulent strains of NDV by having a substitution of arginine (R) for glycine (G) at position 112 at the C terminus of F2 protein. At the N terminus of F1 protein, strain I-2 had a sequence pattern of 117LIG119, which was similar to other lentogenic or avirulent strains and not the 117FIG119 motif of virulent strains.

OIE (6) classifies an ND virus as virulent if it has at least three basic amino acids in the position of residues 113–116 and phenylalanine (F) at 117 (7). An interesting feature, especially with the genome of R2B in relation to the polymerase gene, is that this genome is closely related to the genome of Egypt/2005, which has been designated as a velogenic virus (8). This further substantiates to the fact that there are other virulence factors such as polymerase gene, which also play a major role in determining the virulence of NDV (9).

The host range of the virus is extensive and capable of infecting ~241 species of 27 orders, out of the 50 orders of birds (10). The commonly affected species include domesticated birds–chickens, ducks, turkeys, guinea fowl, Japanese quail, pigeons, and many species of wild birds (10–13). The disease is endemic and has greater impact on rural poultry production in most of Africa, Asia, and Latin American countries (1).

## Implication of ND in Northeastern India

**Figure 1:**
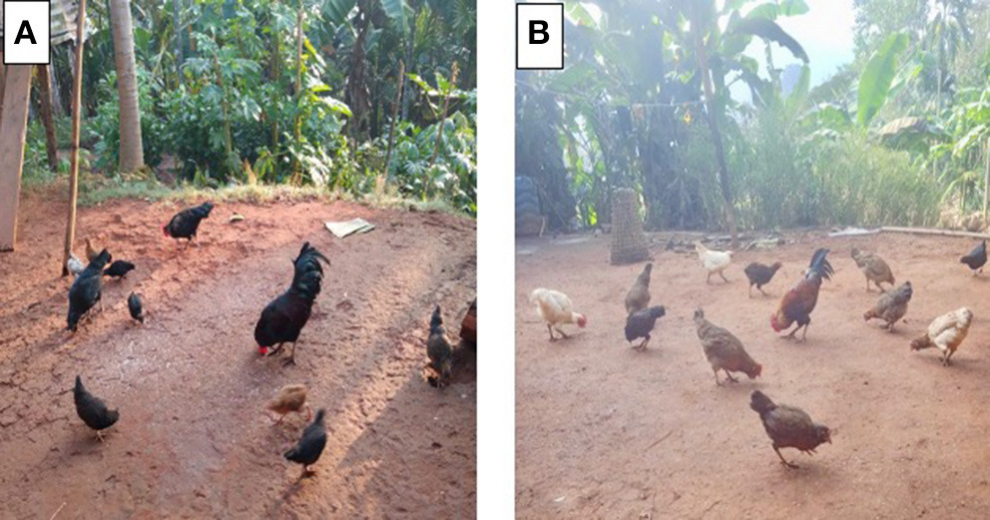
Backyard poultry birds in the villages of Meghalaya in northeastern India comprises of both indigenous germplasm **(A)** and improved varieties **(B)** of poultry.

The northeastern (NE) states comprising of Arunachal Pradesh, Assam, Manipur, Meghalaya, Mizoram, Nagaland, Sikkim, and Tripura have around 7.46% of the backyard poultry population [classified as small extensive and extensive production systems by the FAO (14)] of the country's 217.49 million (19th Livestock Census, 2012) of which more than 60% are indigenous fowls. The total backyard poultry population has notably increased to 317.07 million in 2019 (20th Livestock Census) and increased by 45.8% over the previous census. Backyard poultry production is commonly practiced in rural India. Mostly in the NE region, backyard farming is the practice of rearing indigenous birds by rural folk, with low input and low output. Almost every rural household rears at least 4–5 indigenous birds for meat and/or eggs (Figure 1). The region's geographical features mainly comprised of hilly terrain with scattered plains and valleys. The altitude varies from almost sea level to over 7,000 m (23,000 ft) above mean sea level (MSL) (15) and is suitable for rearing birds under the system of backyard farming. The economic returns from backyard poultry rearing provide an additional income with minimum capital investment in the shortest possible time, simple in operation but it ensures availability of protein in the form of meat and eggs. Furthermore, due to the free range and scavenging nature of the birds, there is the input of manure into the field and is advantageous in pest control too. The colored birds are also used for traditional rituals and auspicious festivals in the community. With the growing population, the need to increase food supplements other than agricultural produces also arises. The national program on integrated farming system approach introduced through the Indian Council of Agricultural Research (ICAR) for livelihood sustenance, and income generation promotes the incorporation of poultry components (chickens, ducks, and related species) in integrated agriculture practiced by rural farmers. The poultry seed project by ICAR-Directorate of Poultry Research introduced improved germplasm varieties of dual-purpose poultry such as Vanaraja, Gramapriya, and Srinidhi for backyard rearing to enhance income and ensure livelihood improvement of farmers. Therefore, smaller units of improved varieties of poultry along with other livestock components and/or agricultural crops or vegetables or fruits have been popularized throughout the region. However, the occurrence of ND, in most instances, has devastating effects and wipes out the entire poultry population in a particular area or village, which results in huge economic losses and makes it difficult to restock flocks.

The other poultry species that include ducks that are largely asymptomatic when infected can spread the virus. The geese are considered susceptible to infection, but the development of clinical disease is variable (16). There have been reports of clinical disease in geese in China with NDV strains of genotype VIId and VIb (17). Specific genotypes of virulent NDV are also maintained in wild birds, such as pigeons and cormorants (18–20). Psittacine birds have also been implicated in the maintenance of NDV infections and in the transmission of disease to poultry species (21, 22). The disease was also reported in vulture (23), emu (24), etc., which indicated the widespread presence of NDV in different bird species and the threat of these carriers to spread the infection across susceptible host which makes it all more difficult and challenging for the prevention and control. There have been reports of outbreaks of ND in vaccinated and non-vaccinated flocks including backyard poultry from various parts of India including Assam, which is of genotypes II and XIII variant (25–30). The broad circulation of NDV in poultry populations led to a significant genetic diversity of the virus and the constant emergence of NDV variants (1).

## Detection, Preventive, and Control Method

Newcastle disease diagnosis is based on the clinical signs, postmortem examination, and serological testing. However, there are other viral diseases that have similar clinical signs, which include avian influenza for which differential diagnosis is required. Therefore, the isolation of virus which is gold standard for virus detection and molecular techniques could be used to detect species-specific genes to confirm NDV. Given the clinical and economical relevance of NDV to the poultry industry and the broad use of vaccines worldwide, sequencing and phylogenetic analysis become the methods for the characterization of NDV strains circulating in the field (1). The sequencing of viral genome allows comparisons of the genetic relatedness of different isolates, which will enable to track NDV evolution and genetic diversity from various host species. However, timely diagnosis is lacking in field conditions. The rural veterinary infrastructure is minimal without the support of established laboratory facilities to aid in diagnosis.

**Figure 2:**
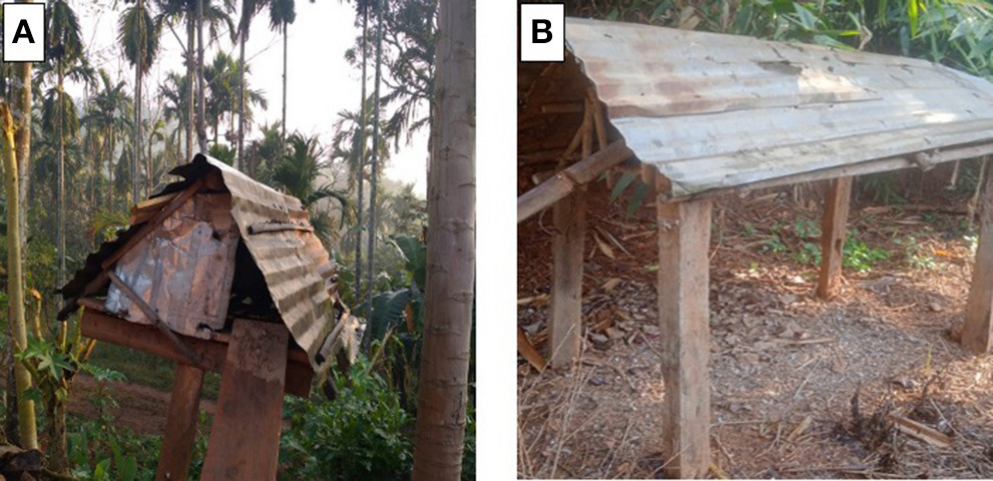
Representative photographs **(A,B)** of rural backyard poultry sheltering structures in villages of Meghalaya state in northeastern India.

General approaches to ND control program are vaccination in combination with appropriate biosecurity measures. But vaccinating the birds and observing biosecurity protocols in the backyard farming system poses a challenge since the birds are let loose into the surrounding of the house and scavenge their food from it. Most of the farmers do not have proper housing (Figure 2) but only makeshift bamboo pole or trees for cooping at night, thereby making it difficult to handle or monitor the health status. ND vaccines are available as inactivated or live vaccines (Table 2). For prophylactic use, the lentogenic strains of NDV of chick embryo origin, such as B1, La Sota, and F, are commonly used as live vaccines. In addition, mesogenic strains – Komarov and R2B – are still used extensively in many Asian countries, which include India, for evoking stronger immune response in susceptible birds (31). The backyard poultry-like layer birds require the vaccination program to provide protection for longer periods as compared to the broiler birds. The low virulent strains (B1 or LaSota) are used as a live attenuated vaccine for 1-day-old chicks and the same has been given as a booster to 14-day-old birds. Thereafter, strain R2B is used for the vaccination at the age groups of 8 weeks or older birds, especially in areas having a high risk of NDV outbreaks (32). Most often, similar strategies are used for control of NDV across other South-East Asian countries. However, the main challenge in ND control is NDV vaccination, which is not practiced or properly documented in backyard poultry in rural areas. One of the main constraints for not practicing vaccination is the commercial vaccine packaging comes in bulk doses whereas the number of birds per household is generally low, which results in vaccine wastage and often prevents the farmers from procuring the vaccine. Also, they have to maintain the appropriate cold chain storage, which is subjected to breakdown due to rampant irregularities in the power supply. Moreover, outbreaks in many areas are largely neglected with no proper containment of infection, which leads to the spread and spillover of NDV outbreaks that have been reported.

**Table 2:** Characteristics of Newcastle disease vaccines [Young et al. (7)].

**Type**	**Inactivated**	**Live mesogenic**	**Live lentogenic**	**Live avirulent**
Immunogenicity	Very good	Good	Moderate	Moderate
Thermostability	Moderate	Poor	Poor	Very good
Production	SPF eggs	SPF eggs (sometimes)	SPF eggs (sometimes)	SPF eggs
Route of	Injection	Injection	Eye drop	Eye drop, feed
Administration			drinking water	drinking water
Transmissibility	n.a.	Yes	Yes	Yes

The administration of NDV vaccines is the primary tool used to prevent the clinical disease. Though it does not provide sterilizing immunity, the levels of viral shedding can be reduced 100-folds with proper vaccination ensuring the decrease in the amount of virulent NDV secreted into the environment, an additional benefit that is rarely considered in control strategies (1). One of the important approaches in NDV preventive measures is herd immunity. Virulent NDV is significantly less likely to occur in the flocks with herd immunity compared to flocks without herd immunity and is also important for preventing the spread of virulent NDV (1, 33). Practically, herd immunity is not often achieved in the field condition, and thus, it is required to carefully monitor flock immunity after vaccination. Herd immunity exists when at least 85% of a flock has hemagglutination inhibition antibody titers equal to or greater than 8 to NDV (when using 8 hemagglutination (HA) units per 50 μl of antigen) and is essential for flock protection against ND (1, 34). If a government institution is produced the vaccine locally, a long-term commitment of staff, facilities, and funds is needed to establish and maintain the production. Mechanisms for cost recovery (e.g., a revolving fund) must be established so that proceeds from the sale of the vaccine are returned to the producer to enable timely purchase of eggs, reagents, and consumables needed for ongoing vaccine manufacture.

The control of ND by vaccination should always be complemented by good husbandry, hygiene, and biosecurity. Good feeding and housing will improve the ability of the chickens to mount a strong immune response to the vaccine. Care should be taken to limit the spread of ND from infected birds by control of the movements of people and animals, segregation of sick birds, and correct disposal of infected birds and their remaining. Remembering that vaccinated birds exposed to virulent ND virus may become infected and excrete virulent virus, although they still remain clinically healthy. Such birds may therefore be a source of infection for unvaccinated birds (7).

It is essential to maintain appropriate biosecurity measures to prevent ND in poultry flocks. The biosecurity practices include accurate record keeping, following proper vaccination schedule, and identifying practices that may facilitate the introduction of virulent NDV or practices that lead to the development of stressful conditions that will hinder an optimal immune response (1). The record-keeping practices that include the recording of mortality, necropsies findings, laboratory diagnostic reports, and proper carcass disposal are necessary to assist in the early detection of disease and are crucial in preventing the spread of NDV into multiple locations (1). It is important to note that hardly any biosecurity measures are practiced in the backyard poultry rearing. Sporadic wet markets in villages, huts, or weekly bazaars witness a random increase in selling the live birds whenever disease outbreaks occur in the area likely increasing the threat to spread and spillover of NDV. Other important biosecurity practices involved the exclusion of species such as pigeons, ducks, and other wild avian species that may be the carriers of diseases and control of pests such as insects and mice and minimizing stressful rearing situations. However, in the backyard rearing system, none of the above-mentioned factors have been addressed properly.

Some of the neighboring countries and India being an endemic region for NDV face outbreaks every season despite regular vaccination programs, and it has a huge impact on poultry production including backyard poultry rearing (35). It has been reported that genotypes of NDV apart from genotype II among poultry in India prevail and cause the outbreaks (28, 29) while generally, vaccines used like strain F, R2B etc., belong to genotype II (2). Hence, the commonly used vaccine strains in India need to be re-examined for the scope and better coverage in its use as a prophylactic. Vaccination against NDV although protects against clinical disease, but it fails to protect against virus shedding when challenged with a different genotype virus (2). Though many reasons could be attributed to it, the presence of the etiological agent in the vicinity may always pose a severe threat even to the vaccinated bird population. This gains importance by the fact that the free-roaming birds have an exposure to a wider area in the locality of the backyard poultry. Wild waterfowl are reported to harbor the lentogenic strains and poultry where velogenic outbreaks that are manifested can circumvent the protective attributes of the vaccine, which leads to the persistence of variant strains of the virus (36). Though ND vaccination usually protects the bird from serious consequences of the disease, virus replication and shedding may still occur and remain as a source of infection. Therefore, it is of utmost importance to adequately design the vaccination program that will give the best protective result in terms of clinical protection and in reducing the shedding of the virus in vaccinated flocks (1).

## Suggested Preventive and Control Strategies

### Awareness for Proper Biosecurity Measures

It creates more awareness about the disease among the rural mass by the State Veterinary Line Department, State Agricultural Universities, and ICAR through Krishi Vigyan Kendra's working in the field. Vaccination and an adequate biosecurity regime must ensure that there is a sufficient period of time for the immunity to develop after the birds are vaccinated and before being exposed to the infectious environment. The robust diagnostic system with prompt and accurate diagnosis through laboratory confirmation could help in the control program. Timely and proper carcass disposal by incineration or deep pit burial is important and critical in containing ND outbreaks because virulent NDV can remain viable in the tissue of infected carcases for weeks and become a source of environmental contamination or direct infection to susceptible birds.

### Strengthening the ND Control Program With Viable Vaccine

Newcastle disease vaccines and good husbandry can prevent the disease in areas where conventional vaccines can be kept cold (14). Using thermotolerant ND vaccine is another option for developing countries such as India especially the NE region where the cold chain transport and storage are difficult to maintain. Bensink and Spradbrow (37) recommended the use of thermostable I-2 ND vaccine in developing countries for the protection of village chickens against ND. I-2 ND vaccines have been used in village chickens in Vietnam (38), commercial and village chickens in Tanzania (39), free-range chicken in Uganda (40), broiler chicken in Iran (41), and V4 vaccine in Gambia (42). The I-2 vaccine has also been and continues to be extensively used in Mozambique (43). In India, the vaccination of backyard poultry with thermo-tolerant LaSota vaccine (44) has been tried in three states–Chhattisgarh, Odisha, and Jharkhand. Also, strengthening the existing infrastructure of the State Animal Health Services and mobilizing the services of Veterinary Field Assistants, Community Volunteers and Farmers in community participatory mode for vaccination could help to strengthen the ND control program.

### Modulation of Immune System Targeting Innate Immune Cells

The avian innate immune system presents an interesting opportunity to prevent disease and potentially enhance flock performance. The newly hatched birds are immediately confronted by diverse pathogenic microbes present in the environment, and their adaptive immune defenses in the early days have limited capabilities to combat these pathogens (45). However, innate immune responses provide a degree of protection. The innate immune system recognizes broad features common to the groups of pathogens known as pathogen-associated molecular patterns (PAMPs). The recognition of PAMPs occurs through the pattern recognition receptors (PRRs), which are present on the host cell. Interactions between PAMPs and PRRs drive the innate immune system to respond to a variety of pathogens and begin containment and elimination of the infection (46). One of the most studied PRRs in poultry is the toll-like receptors (TLRs). Engagement of TLRs with their ligands leads to the induction of non-specific host responses that minimize the replication and eventually guides the adaptive arm of the immune system to generate long-lasting immunity against invading pathogens. The studies showed *in ovo* treatment of TLR-7 ligand resiquimod and TLR-9 ligand CpG enhanced protection against infectious bronchitis virus and avian influenza virus challenge exposure in young chicks, respectively. Various studies showed the TLR ligands when used as prophylactic means could elicit antiviral effects and protection in chickens against respiratory viral infections that could be applied for ND.

### *In ovo* Vaccination

The newly hatched birds in comparison with mature birds have revealed differences in intensity and quality of immune response. The functional expression of PRRs and several defense molecules of immune system of embryos and newly hatched birds indicated that innate responses could be modulated effectively at this stage of the development to combat pathogens (45). The current knowledge on the action of the vaccine in conjunction with *in ovo* administration of TLR ligands elicited response, and their immunomodulating ability draws attention to their potential use as the therapeutic agents for the poultry industry and this will be of relevance specially to the backyard poultry where complete vaccination regime and strict biosafety measures were not properly followed. Most of the improved variety chicks were supplied to the farmers from government hatcheries or private firms. Intervention at this production point could well be considered for *in ovo* vaccination against ND. But, given that some backyard birds can live for a number of years, revaccination against ND is required. So, *in ovo* vaccination may protect the birds initially, but the vaccination within households will still be required if the disease is to be successfully controlled. In advanced countries, *in ovo* vaccination has been used for infectious bursal disease, infectious bronchitis, Marek's disease, ND, and others.

### Vaccine Quality

Any vaccines developed to meet the special needs of village chicken farmers in developing countries are to adhere to the quality criteria. To meet these needs, vaccine producers have a responsibility to produce the vaccine that is:

- Safe—will not cause local or systemic reactions when used as recommended by the manufacturer.
- Potent—contains sufficient virus to induce a protective immune response.
- Effective—will protect chickens from virulent ND.
- Pure—free of extraneous micro-organisms and material.
- Easy to use.
- Affordable.

These features define the quality of the vaccine (47), which is considered “the single most important determinant of vaccination success or failure” (48).

To ensure the consistent production of good quality vaccine, the producer must put in place standards and controls covering all aspects of its manufacture and handling. These standards and controls “define the risk or possibility of producing and releasing a product that is worthless, contaminated, dangerous or harmful” (6) and should be determined by local resources and needs. Standards and controls should not be so expensive, demanding, and time-consuming that farmers are unable to purchase the vaccine to protect their flocks.

The principles of quality assurance (QA), good manufacturing practice (GMP), and quality control (QC) define the standards and controls that ensure the production of good quality vaccine and are the foundations of good vaccine production.

Quality assurance includes all the arrangements made to ensure that the vaccine is manufactured to a quality appropriate to its intended purpose—in the case of ND vaccine, the vaccination of village chickens. All the aspects of vaccine production and testing (such as the facilities and personnel, procedures and records, starting materials, product testing, labeling, packaging, and distribution) are considered. QA ensures that the process of vaccine production is uniform and consistent through production procedures and product testing. It ensures that the process of vaccine production is designed, documented, implemented, and furnished with personnel, equipment, and all resources.

Good manufacturing practice is that part of QA that ensures that a product is manufactured in a safe, clean environment, by specified methods under adequate supervision, and with effective quality control procedures.

Quality control is that part of GMP concerned with the taking and testing of samples at each stage of the production process to ensure the safety, purity, potency, efficacy, and stability of the vaccine. QC also ensures that the vaccine is not released until it passes these tests. QC alone will not guarantee the quality of the vaccine; it is better and cheaper to prevent the problems with vaccine quality through good QA and GMP than to rely on tests on the final product!

The procedures and protocols described are the minimum required to ensure the production of I-2 vaccine of good quality, which is suitable for the use in village chickens. Vaccine production and testing protocols should be revised regularly, and staff should be encouraged to refine and improve procedures, so that standards outlined in *OIE manual of standards for diagnostic tests and vaccines* (6) are maintained (7).

## Conclusion

Backyard poultry rearing constitutes an important component in the livelihood of marginal farmers, especially in the NE India. Many times, these farmers face difficulties in accessing vaccine against specific disease such as ND due to varied reasons—dose size of vaccine, cold chain maintenance of conventional vaccines, the lack of participatory approach, and awareness of ND in the community among the poultry farmers. Therefore, creating more awareness involving all the stakeholders and opting for small dose size thermotolerant ND vaccine might be one option to control this disease. Strengthening the diagnostic capabilities would elucidate and identify the circulating strain of NDV in the region. This would substantiate the choice of type of vaccine for ND control program. Also, a means to boost the innate or non-specific immune system of birds to combat pathogens in the local environment together with vaccination and better biosecurity measures potentially will serve the poultry farming in controlling morbidity and mortality due to ND. However, improved understanding of the birds' immune system and functionality is critical for safe interventions to provide effective long-term protection against pathogens and to devise more efficacious disease control strategies.

India being an endemic country for NDV, outbreaks of the disease occur every passing year. It has also been recently reported about the persistence of genotype IV strains in India. The results of the study reported herein indicate that the conventional vaccines such as strain F and R2B belong to genotype II. The reports suggest that conventional vaccination against ND virus prevents clinical disease and, compared to non-vaccinated birds, reduces the amount of virus shed but virus shedding may still be higher when challenged with ND virus of a different genotype (49). Considering these facts, the commonly used vaccine strains in India need to be evaluated further for their applicability in the field and protection potential (50). Therefore, it can be stated that concerted effort by the poultry farmers, animal health workers, vaccine manufacturers or suppliers, and policymakers is needed to bring change in control and prevention of this dreaded disease in backyard poultry in the northeast region of India.

## Author Contributions

The manuscript was written by KP with opinion and inputs added by AS. All authors read and agreed upon the content of the manuscript for submission.

## Conflict of Interest

The authors declare that the research was conducted in the absence of any commercial or financial relationships that could be construed as a potential conflict of interest.

## Publisher's Note

All claims expressed in this article are solely those of the authors and do not necessarily represent those of their affiliated organizations, or those of the publisher, the editors and the reviewers. Any product that may be evaluated in this article, or claim that may be made by its manufacturer, is not guaranteed or endorsed by the publisher.
